# “It's not just teaching for the sake of teaching anymore”: a quality improvement project for online instruction in a hospital library

**DOI:** 10.29173/jchla29771

**Published:** 2024-08-01

**Authors:** Melanie Anderson, Caleb Nault, Raluca Serban

**Affiliations:** 1 Information Specialist, Library and Information Services, The Institute for Education Research, University Health Network, Toronto, ON; 2 E-Collections and Discovery Librarian, Library and Information Services, University Health Network, Toronto, ON; 3 Library Technician, Education Coordinator, Library and Information Services, University Health Network, Toronto, ON

## Abstract

Medical libraries had to swiftly change how instruction services were provided in response to the COVID-19 pandemic. This article describes a Quality Improvement (QI) project to assess one hospital library’s move to virtual instructional services. Information was gathered via a survey to Canadian hospital library workers and a focus group with internal staff with instructional responsibilities. Moving to online instruction increased audience attendance and garnered positive feedback, however library instructors report experiencing uncertainty about quality and effectiveness of online instruction. The article concludes with a description of planned actions to improve online instructional services in an urban research and teaching hospital library environment.

## Introduction

University Health Network (UHN) is a large system of teaching and research hospitals and an education institute located in Toronto, Ontario, Canada. A team of 13 staff at Library and Information Services (UHN Libraries) supports the wide-ranging professional development and information literacy needs of the clinicians, researchers, staff, and learners who work at UHN. As an adaptive response to the COVID-19 pandemic, UHN Libraries translated its instructional services from in-person workshops to synchronous virtual sessions. Library instructors applied their existing knowledge and experience of developing and leading in-person workshops to teaching in an online environment. Despite a dramatic increase in attendance and sparse but positive client evaluations, instructors expressed uncertainty about how well their skills transferred to the new format and questioned whether the service could be improved. To address these concerns about the new online instruction services, UHN Libraries engaged in a Quality Improvement (QI) project.

QI systems and processes have long been utilized in health care settings to evaluate and improve service delivery [1]. Libraries in health organizations have also undertaken QI to assess the quality of library services and have used a range of qualitative, quantitative, and mixed-method practices from benchmarking, interviews, and questionnaires [2] to proprietary methodologies like Six Sigma [3] and Lean management techniques [4]. QI has been used to examine and improve various library services, programs, and workflows, including standardizing mediated literature searching [5], updating reference interview practices [3], reorganizing physical library space for optimized service delivery [4], and expanding information services within a clinical librarianship program [6]. This article describes a QI project undertaken to reconceptualize and transform UHN Libraries’ instructional services and concludes by exploring recommendations to enhance online instruction in an urban research and teaching hospital library environment.

## Description

### The problem

Following the move to remote work in response to the pandemic, library instruction sessions were translated into virtual events that were staffed by three library members: the instructor, a chat monitor, and a reserve instructor. In January 2021, an Instruction Team comprised of library staff with teaching responsibilities and led by a library technician in the role of Education Coordinator began overseeing the assessment, updating, and improvement of existing workshops, and the development of new content. As a result, a repertoire of online workshops started being offered synchronously twice a month. Moving to online instruction resulted in a dramatic increase in attendance and sparse but positive client evaluations, however library instructors expressed uncertainty about how well their skills transferred to this new format and questioned whether the service could be improved.

### Aims

The Instruction Team sought to learn about the approaches used by other hospital libraries for online instruction and the circumstances that facilitate or complicate these approaches. The team was also interested in any existing best-practices for online instruction in hospital library settings, and in the strengths and weaknesses of changes made to UHN Libraries’ instructional services.

### Planning

To achieve these aims, a subcommittee of the Instruction Team embarked on a QI project. QI is a structured but adaptable process aimed at assessing and evaluating programs or services, identifying current practices and opportunities for change, and rapidly implementing new knowledge into practice [7]. The QI process at UHN involves submitting a QI project submission form and supporting documents to the Quality Improvement Review Committee to confirm that the project is QI and does not need to be directed to the Research Ethics Board, that the project meets all ethical and legal responsibilities, and that the project minimizes or eliminates risks to the project team, participants, and UHN [8].

The plan approved in November 2021 outlined an information gathering process that included a search of the literature, a survey distributed electronically to hospital libraries across Canada, and a semi-structured conversation with UHN library staff with instructional responsibilities. Further, internal workshop statistics for in-person and virtual workshops would be analyzed. Approved avenues for dissemination included sharing the results internally with colleagues and externally with peers from other institutions via conference presentations and a publication.

### Literature

Documents produced by health library professional associations mention instruction as a standard library service and area of librarian competency. The CHLA Standards for Library and Information Services in Canadian Health & Social Services, under the third standard “Services”, specifies “[e]vidence-based practice/information literacy training” as part of the “minimum level of library services” [9]. Beyond stipulating instruction as an essential service, however, the Standards do not provide guidance about what such training could or should entail. The Medical Library Association (MLA) identifies “Instruction & Instructional Design” as a librarian competency, stating that “[a] health information professional educates others in the skills of bioscience, clinical, and health information literacy" [10]. The document indicates that competency in instruction means developing curricula based on instructional design principles, using learner-centered approaches and leveraging “innovative instructional and communication methods and technologies” [10-11]. Although both professional associations declare instruction as an area of librarian competence and essential service, neither provide further information to guide practical applications.

The literature describing online instruction initiatives in libraries primarily reflects academic library contexts. Approaches to online instruction include course-integrated synchronous one-shot sessions and asynchronous multi-part tutorials [11,12]; a stand-alone library workshop series hosted on academic learning management systems [13]; information literacy courses delivered via external online classroom platforms [14]; synchronous sessions using web-based video conferencing platforms [15] and mobile devices [16]; and asynchronous online instructional modules and massive online open courses [11,17]. The abrupt turn to remote work during the pandemic resulted in some academic health science libraries providing virtual consultations, drop-in office hours, self-paced modules, and workshops covering newly relevant topics [18]. Instruction in hospital libraries differs from academic environments, however, because the client population is a mix of professionals and learners with restrictive schedules and unpredictable shift work who may work at multiple sites and who have diverse continuing education and urgent point-of-care information needs. These circumstances can present challenges for developing and delivering relevant educational materials in hospital libraries [15,19,20]. Hospital librarians and library staff have deployed creative solutions to address these client-specific information needs by providing in-person lunch time office hours on a unit [21] and participating in morning rounds [22], but there are few published examples of hospital libraries bringing instructional services to their clients via virtual means.

### Data collection

#### Survey

A survey was developed by the authors to gather information from Canadian hospital libraries about delivering online instruction. The survey was reviewed by peers and consisted of 27 questions that incorporated a mix of multiple choice and open-ended answers (see Appendix 1). The questions covered topics such as online instructional services policies and procedures, online instructional design and modalities, and barriers and facilitators to delivering online instruction. The survey was disseminated through Canadian health science information listservs in December 2021 and January 2022, followed by targeted outreach in March 2022 to underrepresented regions of Canada, including provincial health library associations and key individual contacts. Eligibility criteria required that the survey respondent work in a hospital library in Canada that offered online instruction. By survey close on March 31, 2022, 26 eligible responses had been received.

#### Guided conversation

After collecting and analyzing the data from the survey, in April 2022 another phase of information gathering focused on the perspectives of instructors from UHN Libraries. A total of 10 open-ended questions guided a group conversation that explored topics such as time available to dedicate to teaching; training received around designing and providing instruction; thoughts on the library’s existing instructional services; and barriers encountered in planning and delivering online instruction (see Appendix 2). Two of the authors led the conversation with seven librarian instructors.

## Outcomes

### Approaches to online instruction

Most of the survey respondents were relatively new at providing online instruction. 57% of the survey respondents indicated that their libraries either started online instruction or made big changes to expand their online instruction because of the pandemic. This response reflects a significant increase to the results reported in a 2018 publication, wherein 35% of respondents stated that their libraries provided online instructional content [19]. Similar to other libraries, the pandemic appears to have catalyzed a change in instruction delivery for hospital libraries in Canada.

Almost 77% of survey respondents are offering workshops in similar formats to those at UHN Libraries. These take the form of lectures and live demonstrations and include interactive components such as question and answer periods, polls, and breakout rooms. Survey respondents also offer asynchronous online training in recorded demonstrations, interactive modules or tutorials, and worksheets and handouts.

### Barriers and facilitators to providing online instruction

**Fig. 1:**
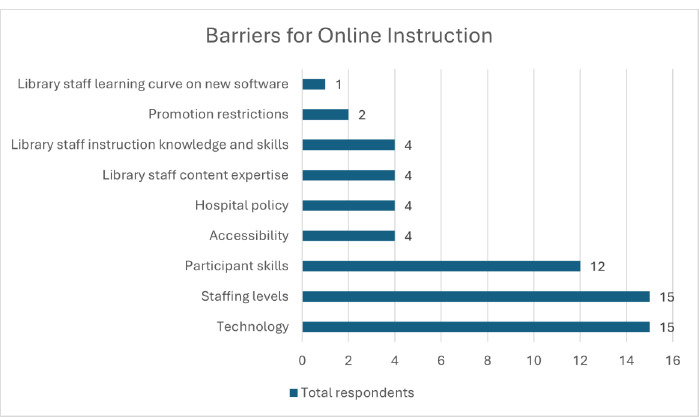
Barriers for instruction identified by survey respondents (see Appendix 1)

The most common barriers for survey respondents were lack of time, inadequate staffing levels, and the perceived underdeveloped technological skill levels of both staff and workshop participants (Figure 1). This echoes the 2018 results of Sandieson & Goodman’s survey of online instruction in hospital libraries [19].

The survey respondents noted that improved access to innovative technologies can facilitate providing instruction by enabling remote learning and multiple modes of instruction. Additional enablers mentioned by survey respondents included having prior knowledge and experience with online instruction, familiarity with content, access to in-house education experts and professional development or continuing education. Other survey respondents mentioned being open to trying new things, having a best practices manual for workshop development, collaborating with other hospital departments, and having an “Education Lead" guiding the process as enablers to providing online instruction.

Common across the literature, survey responses, and guided conversation with UHN instructors is the notion that library workers routinely receive minimal-to-no training in teaching practices [23–28]. 15% of the survey respondents identified a lack of teaching skills or knowledge as a barrier to online instruction. Others also mentioned the further limitation of insufficient time for professional development or training: “I know that some tools could be stimulating, motivating, have a ludic approach, but I have no time to acquire practical skills with these.”

While one UHN library instructor had received formal training in instruction, the rest had not: “I would say that my first library job, I was mentored by other librarians for instruction, but I never received any formal training. Just kind of to do presentations in grad school and then when I started a library job suddenly had to teach, which was very frightening.”

Across the conversation with instructors and survey responses, it was noted that it is vital to provide time and access for continuing education in many forms. Examples drawn from the survey include sharing experiences in communities of practice, time and potentially funding to attend webinars, tutorials, or conferences, and providing guiding documents.

### Strengths and weaknesses of UHN Libraries’ Instruction Services

**Fig. 2:**
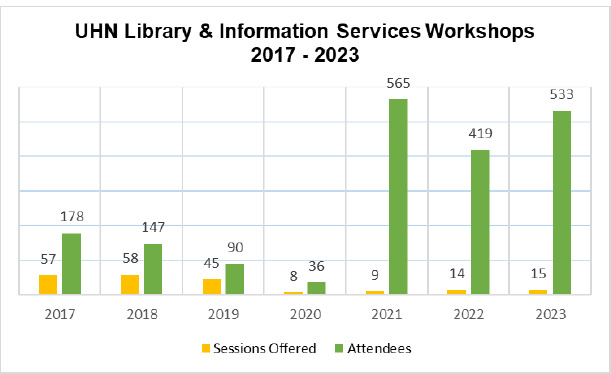
The number of UHN Libraries' workshops offered and attendees from 2017-2023

At UHN Libraries, attendance numbers and attendee feedback indicated that the online instruction program initiated due to the pandemic was a step in the right direction. Before the pandemic, instructional services at UHN Libraries centered on a roster of eight workshops, all delivered in-person three to five times a month at each of the five UHN library locations. These voluntarily attended, stand-alone sessions were primarily taught by hospital librarians and were updated infrequently by the individual instructor. From January 2017 to March 2020, UHN Libraries taught 168 workshops that were attended by 451 clients, for an average of less than three clients per scheduled session. After shifting to exclusively online synchronous instruction sessions, an average of 40 library users have been attending each workshop (see Figure 2). Post-workshop feedback from attendees was positive, with clients selecting an average of 4.5 out of 5 stars in answer to the question “how valuable was this session to you?” for sessions offered in 2023.

In the guided conversation with UHN library staff, the instructors confirmed that establishing an Instruction Team was a significant improvement to earlier processes for in-person workshops because it enhanced the planning, design and delivery of sessions: “Before, we would teach things just to teach things, now we have a lot more thought and consideration into what we're teaching. ... It's not just teaching for the sake of teaching anymore and I think people are enjoying it more.”

However, UHN library instructors felt that continuing to offer the same workshops, whether in-person or online, was repetitive: “I've been here for 10 years, and I've been teaching the exact same thing for 10 years. I don't know about you but like after a while it's not very interesting. It's easy! ... but it's not terribly fun.”

Another area where UHN instructional services could be improved is instructor confidence. The culmination of a lack of training, inability to interpret audience body language due to online delivery, and limited feedback from post-workshop evaluation leads to staff feeling uncertain about their teaching skills, and consequently, whether improvements are desired or necessary. One instructor commented “I often worry that I could be better or I could be doing something different. Or maybe there's better ways to communicate things or get things across.”

## Discussion

Based on the information gathered from the QI process, the Instruction Team has prioritized three areas to improve in the immediate future: better client and peer feedback options, more diverse topics and modalities, and increased engagement with professional development opportunities.

A voluntary peer feedback system has been designed to support instructors in building their skills and confidence. At the monthly Instruction Team meeting, time is set aside for a debrief using peer and instructor feedback, attendance information, and attendee evaluations to discuss successes and potential improvements for future workshops. This system will be assessed at the conclusion of a full instructional program in 2025.

To counter instructor disengagement and reach busy audiences, the Instruction Team is working to expand beyond the roster of hour long, one-off workshop sessions to other formats. In 2023 and 2024, several shorter videos, an online interactive tutorial and a new workshop series were created. Assessments for these initiatives will incorporate both quantitative and qualitative feedback from users and the Instruction Team.

The instructor training needs of the library team are an important aspect of the library’s existing support for continuing education. There are professional development opportunities available through professional associations and UHN’s Clinical Education portfolio. Leveraging the expertise of the institution’s Clinical Teaching and Learning Development team, inviting guest speakers to present on specific instruction skills, developing an internal community of practice, and developing an online instruction guidance document are all under consideration for future initiatives.

UHN Libraries' existing online instruction services are succeeding in many ways, not the least of which has been the improved efficiency of reaching so many more learners with many fewer sessions. In the absence of existing published best practices on online instruction in hospital libraries, the structured QI process generated many ideas for potential improvements to the design and delivery of online instruction and confirmed that libraries in hospitals across Canada are encountering similar barriers around time, capacity, technology, and training.

## Appendix 1 – Survey email

### Online Instruction in Hospital Libraries in Canada: UHN Quality Improvement Project

Hello and welcome,

You are being invited to complete a survey for a UHN Quality Improvement (QI) project for UHN Libraries & Information Services on *Online Instruction in Hospital Libraries in Canada*. This survey is being done to consult communities of practice to identify areas for improvement in instructional services. The information you provide will be used to determine instruction service gaps. It will also be used to develop an instructional services policy.^[1]^

Taking part in this survey is voluntary. Information you provide will only be seen by information professionals (librarian, library technicians) employed at UHN Library and Information Services. Others within UHN and outside of UHN will only see a summary of the overall information collected. Your responses will not be linked to your name or personal information in any way and will be stored separately from your personal information. Survey responses will be stored on the Springshare data server in Canada for one year. If results of this survey interview are published or presented at meetings, your name and other personal identifying information will not be used, and your responses will not be linked to your name or personal information in any way.

If you have questions about this QI project, please contact Melanie Anderson (melanie.anderson@uhn.ca). If you have questions about your rights as a participant in a UHN Quality Improvement Project, please contact the UHN Quality Improvement Review Committee (QIRC) at ---@uhn.ca. QIRC is a group of people who oversee the ethical conduct of QI projects; they are not part of the project team.

Thank you for your participation!

^[1]^ See TCPS 2 (2018) – Chapter 5: Privacy and Confidentiality Section D for a description of consent and secondary use of data, if needed: https://ethics.gc.ca/eng/tcps2-eptc2_2018_chapter5-chapitre5.html

Survey Questions

1. Do you work in a hospital library? Y/N
2. Does your library offer instructional services? Y/N
3. What types of instruction does your library offer? (Select all that apply)
  - Workshops
  - Classes/courses
  - Informal/as-requested training
  - Self-directed learning materials
    - eLearning modules
    - Handouts/instruction sheets
    - On-demand or pre-recorded videos
  - Other:
4. Does your library offer online instruction? Y/N
5. When did your library start offering online instruction? (text box)
6. If your library moved instructional services online, have there been changes to your:
  - Process (text box)
  - Teaching methods (text box)
  - Evaluation practices (text box)
  - Promotion (text box)
  - Attendance, attendee satisfaction, number of workshops offered (scale - increase, stayed the same, decrease, not applicable)
7. Does your library have an instructional services policy?
  - Yes
  - No
  - Unsure
8. How do you determine what workshops to offer? (text box)
9. Do you collaborate with departments external to the library to create instruction material? Y/N
10. How many people are involved in planning for instruction?
11. How many staff work in your library?
12. What barriers have you encountered in planning and delivering instruction for your library?
  - Technology (platforms, tools like mentimeter, canva, etc., internet access etc.)
  - Hospital policy (privacy, etc.)
  - Accessibility
  - Staffing levels
  - Participant skills (audience comfort with technology?)
  - Library staff content expertise
  - Library staff instruction knowledge and skills
  - Other: (text box)
13. What has helped you provide online instruction? (text box)
14. How many people are involved in delivering each instruction session in the library? (text box)
15. What instruction methods do you use? Select all that apply.
  - Hands-on
  - Group work / breakout rooms
  - Flipped classroom <<hover over question mark to read description>> Introduce learning material before workshop and during session spend the time discussing with peers and problem-solving activities facilitated by Information Specialist
  - Interactivity (polls, word clouds, quizzes, etc.)
  - Feedback <<hover over question mark to read description>>
  - Other: (text box)
16. What tools are you using for online instruction: (all text box answers)
  - Platforms (Zoom, MS Teams, Webex, Google Meet etc.)
  - Interactivity tools (Mentimeter etc.)
  - eLearning software
  - Audio/video software/tools
  - Visual design tools (Canva, Visme, etc.)
  - Publishing (LibGuides, YouTube, Wordpress, etc.)
  - Other:
17. What style of workshops does your library offer: (check as many as apply)
  - One-off/single session
  - Skill level progression (basic to advanced)
  - As a series (3 in-depth sessions on systematic review methods)
  - Other
18. Which tools have you had the most success with? (text box)
19. What workshops does your library offer? (text box)
20. How do you evaluate your online workshops? (text box)
21. Is there anything else you’d like to tell us about online instructional services? (text box)
22. Is your library single site or multiple sites? (select one)
23. In which province or territory is your library located? (drop down or select one)
24. How would you describe the size of your organization? (text box)
25. What kind of hospital is it? Check all that apply
  - Teaching
  - Community
  - Research
  - Rehabilitation
  - Other:
26. Who does your library serve?
  - Hospital staff
  - Patients
  - Researchers
  - Other:

**Thank you!**

## Appendix 2 – Questions for the UHN library & information services guided conversation

1. What do you consider to be instruction?
2. Did you receive training on instruction? How long have you been teaching? How comfortable do you feel with instruction?
3. How much of a priority do you think instruction should be?
4. Do you have concerns about our instruction services?
5. Aside from workshops, what other types of instruction are you doing? Has anything changed?
6. Are you satisfied with the range of topics we offer workshops on? Are you concerned that we’re missing anything?
7. What barriers have you encountered in planning and delivering instruction
8. What has helped you provide online instruction?
9. Is there anything that could support your teaching development? Skills development?
10. Is there anything else you’d like to tell us?
